# Augmented Reality for People with Low Vision: Symbolic and Alphanumeric Representation of Information

**DOI:** 10.1007/978-3-030-58796-3_19

**Published:** 2020-08-10

**Authors:** Florian Lang, Albrecht Schmidt, Tonja Machulla

**Affiliations:** 8 Institute Integriert Studieren, JKU Linz, Linz, Austria; 9 Jack Baskin School of Engineering, UC Santa Cruz, Santa Cruz, CA USA; 10 Dipartimento di Meccanica, Politecnico di Milano, Milan, Italy; 11 Support Centre for Students with Special Needs, Masaryk University Brno, Brno, Czech Republic; LMU Munich, Frauenlobstr. 7a, 80337 Munich, Germany

**Keywords:** Augmented reality, Low vision, Symbolic and alphanumeric representation

## Abstract

Many individuals with visual impairments have residual vision that often remains underused by assistive technologies. Head-mounted augmented reality (AR) devices can provide assistance, by recoding difficult-to-perceive information into a visual format that is more accessible. Here, we evaluate symbolic and alphanumeric information representations for their efficiency and usability in two prototypical AR applications: namely, recognizing facial expressions of conversational partners and reading the time. We find that while AR provides a general benefit, the complexity of the visual representations has to be matched to the user’s visual acuity.

## Introduction

A large percentage of people with visual impairment (VI), including those who have been diagnosed as legally blind, have residual visual functions [6], such as intact peripheral vision with a central scotoma. These individuals often prefer using vision to other modalities – especially, when visual information better supports the current task (e.g., navigation, identification of objects), or when audition and touch are occupied by other tasks. Hence, there is a large potential for the research and development of assistive solutions that provide adapted visual feedback.

A promising approach is the use of augmented reality (AR), presented through head-mounted displays (HMD), to enhance or substitute degraded visual information. When compared to traditional assistive solutions, such as CCTV or optical magnifying glasses, HMDs have the advantage of being mobile and hands-free. Also, AR glasses will likely become an everyday device in the next decades, reducing the stigma that is often associated with the use of assistive devices [7]. A number of recent studies have demonstrated possible applications for AR, such as enhancing text for reading [10] or poorly-lit ledges [13]. There is comparably less work regarding how AR goggles could be used to substitute degraded or imperceptible visual information [14]. In the present paper, we investigate how different representation formats of such substituting information influence the efficiency and usability of AR assistance.

Missing visual information can be substituted by recoding it into a visual format that is more readily perceived by a user with a visual impairment. Here, we distinguish between two basic types of representations: alphanumeric and symbolic. Alphanumeric representation refers to the recoding of visual information into words and numbers. Symbolic representations can be analogues to the information they are substituting for, e.g., when they are pictorial in nature. Alternatively, they can be visually simple or abstract symbols that are easy to perceive and recognize (e.g., a red square, a blue circle etc.) but have little resemblance to the information they stand for. The present research evaluates symbolic and alphanumeric representations of information in AR. For this, we identified two viable use cases from prior interviews with persons with VI: i) reading the time off of conventional watches, and ii) identifying the emotional expression on the face of a conversational partner. Accordingly, we developed two assistive AR functions: one to augment a physical watch with either an analogue/symbolic or a digital/numeric representation of time and another one that overlays the face of a conversational partner with an additional symbolic or textual representation of their current facial expression (angry, sad, happy). Our results show that AR can provide a recognition benefit in both use cases and is generally well-received by persons with VI.

## Related Work

There is some prior work that we considered in the design of our study, in particular regarding the cognitive processes associated with symbolic and alphanumeric representations as well as the use case of augmenting emotional expressions. We are not aware of any scientific work on augmenting clocks visually, most likely due to the fact that there are alternative solutions such as using zoom or audio output on smartphones. However, especially older participants who lived most of their live without any VI and are now faced with gradual vision loss indicated to us that they would like to continue using wristwatches. Here, AR can provide a simple solution without the necessity of having to modify one’s life-long routines, e.g., display a large virtual clock in AR after the user performs the typical gesture of looking at their wristwatch. In contrast, using a future AR device should not require that much of a behavioral adaptation, as many older users already wear normal glasses.

*Symbolic vs Alphanumeric.* Alphanumeric representation of information is very common, especially in user interfaces. Thus, for most users, information processing can be expected to be very efficient without much practice due to a high level of familiarity with the task. Nevertheless, there are two main reasons to evaluate the feasibility of providing information in a symbolic format. First, alphanumeric representation typically requires a higher visual acuity compared to symbols, especially if the latter are sufficiently simplified. Second, information in written form takes longer to comprehend than pictorial information [8] and can require more attentional resources to process, leading to cognitive capture [12].

*Use Case Emotions.* Persons with visual impairments often have difficulties perceiving nonverbal conversational clues, such as facial expressions [2], making social interactions challenging. To support a user during a conversation, an assistive system has to be able to first extract and process these clues and then convey the information in an adapted format to the user. For both steps, relevant prior work exists. Recognising facial expressions from images is a long standing challenge in computer vision [3]. Handcrafted algorithms run in real time and exceed 70% accuracy on mobile devices such as smartphones [11] and reach 97% accuracy on stationary setups with controlled lightning conditions [5]. A neural network can provide more robustness to image properties or fuse multiple modalities [4]. For presenting the information, several researchers have proposed solutions for people with VI using audio [1] or tactile [2] feedback. Zuniga et al. proposed a system providing visual feedback [14]. They presented colored patches in a smartphone-based virtual reality to indicate the current facial expression of a conversational partner as well as a miniature image of the face in the visual periphery, albeit without evaluation with their target group. Here, we test different symbolic and alphanumeric representations for their efficiency to communicate emotional expressions.

## Prototypes and Real World Feasibility

We are currently implementing a prototype for emotion recognition and communication using the Microsoft HoloLens. When a user indicates the start of a conversation, computer vision is used to detect faces and determine facial expressions. In case multiple faces are detected, the largest or most centered face is selected. The information is recoded into an alternative visualization (for details see below) and displayed to the user. The user can adjust the details of this visualization such as its size, position, brightness, and type. The augmentation appears either relative to the screen or to the detected face.

**Fig. 1.**
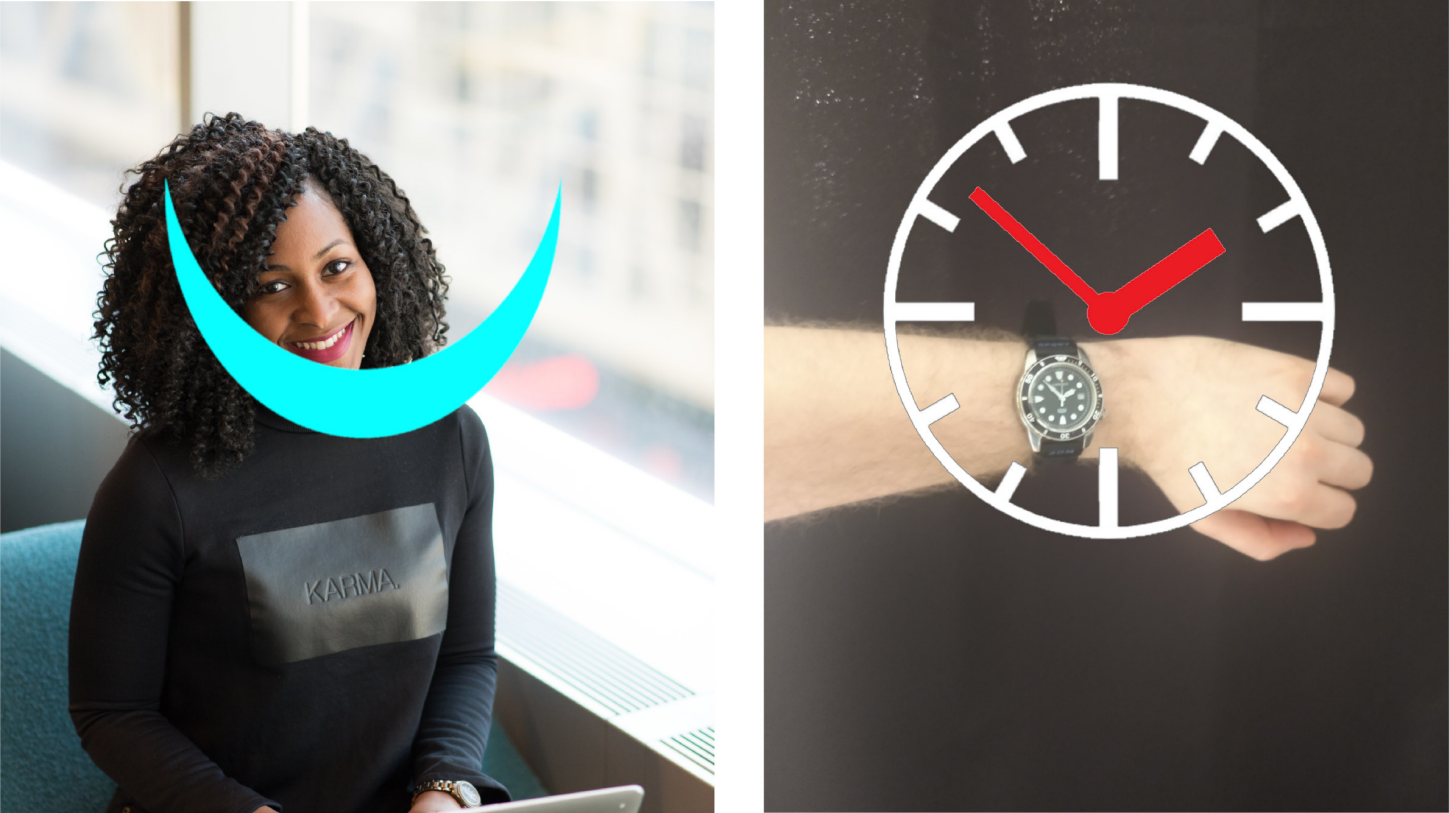
The figure shows the functionality of the two systems in a real world setting. In the left image the facial expression of a conversational partner is augmented to indicate the smiling (Source without augmentation: WOCInTechChat.com (www.flickr.com/photos/wocintechchat/25392590663/ License: www.creativecommons.org/licenses/by/2.0/)). The right side shows a wristwatch which is augmented by the HoloLens. In both cases our system provides a large and simplified representation of information.

Similarly, clock faces can be recognized [9] and augmented. Pilot tests with two individuals with VI showed that positioning the augmentation around a wristwatch is cumbersome, as the watch has to be placed in the comparably narrow field of view of the HoloLens. This means the user had to either tilt their head down unnaturally or lift their arm high in front of their face. Therefore, we pursued an ego-centric approach where the augmentation is always displayed inside of the field of view. We used a smartwatch to trigger the augmentation using a wake-up gesture or a tap on the display. Therefore, users used to regular wristwatches do not need to learn a new gesture. Again, the user can set the type, color, brightness, and position of the augmentation according to personal preferences. A visualization of both prototypes is shown in Fig. 1.

## Methods

We used an iterative approach including feedback from persons with VI to design and develop several early prototypes of the two assistive functions and the different information representations. In the following, we report a formal evaluation of the current prototype.

**Participants.** Participants were reimbursed with 12 EURO/hour. Three individuals with VI participated in the study (P1: 20-year-old male, severe binocular vision loss, 0.04 visus; P2: 24-year-old female, binocular low vision with good color vision, 0.14 visus; P3: 81-year-old female, central vision loss, 0.17 visus). P1 and P2 tested both assistive functions. They had no prior experience with AR devices. P3 tested the clock augmentation and provided feedback on an earlier version of the emotion representations; she has had extensive experience with the HoloLens due prior testing of other research prototypes at our institution. Unfortunately, data collection was interrupted by the Covid-19 pandemic. Although, the generalizability of the current dataset may be limited, we are convinced that it provides constructive insights for future empirical studies and interface design.

**Apparatus.** The AR system is implemented in Unity 3D with the Mixed Reality Toolkit from Microsoft and deployed to a HoloLens. A computer—connected via a dedicated WLAN Router—using the virtual input functionality from the Microsoft HoloLens App controls the application. We implemented the app on the wristwatch in Java (Watch OS 2/Android). We use the touch screen of the devices as input.

**Study Design.** Our primary aim was to test different information representations. Thus, we did not use the full systems described in the above “Prototype” section. This simplified the study setup and removed potential confound variables. In each study, we only display the augmentation, i.e., show the visualization for an emotion without a corresponding conversation and a time of day without the triggering gesture.

**Fig. 2.**
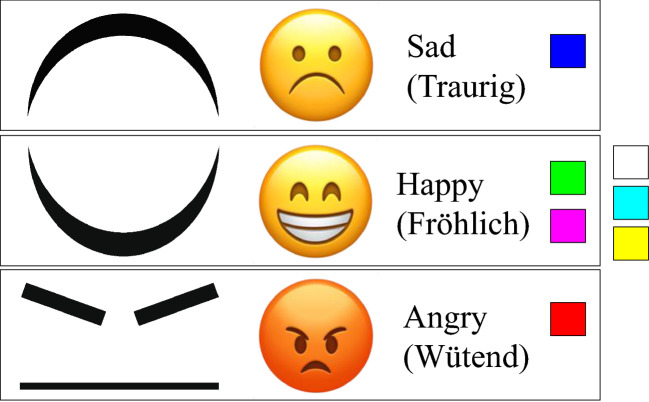
The figure shows the different representations for sad, happy, and angry from top to bottom and from left to right in the conditions Abstract, Emoticons, Text (English and German), and Colors. The three colors on the far right were not mapped to any emotion by any of the participants.

Both studies consist of four conditions, presented in randomized order for each participant to mitigate order and training effects. In the emotions study, one of four different representations was presented in each condition, namely: one textual (alphanumeric) representation, henceforth termed “Text”, and the three symbolic representations Emoticons, Abstract, and Colors (see Fig. 2). In each condition, we displayed representations of the three emotions happy, sad, and angry (following [14]). Each condition consisted of 9–15 training and 15 test trials. Maximum font size of the text was chosen such that the longest word (in German) fit in the field of view of the HMD (approx. visual angle of 17$^\circ$ vertical and 34$^\circ$ horizontal). In the Emoticons condition, we used default emoticons from IOS, as had been suggested by P3 in a prior iteration of the design process. In the Abstract condition, we simplified the emoticons by only displaying the mouth, and for ‘angry’ the eyebrows. In the Colors condition, we asked each participant to select their own preferred three colors (from a set of seven), one for each emotion, for the reason that there is no wide-spread consensus on the association between colors and emotions.

**Fig. 3.**
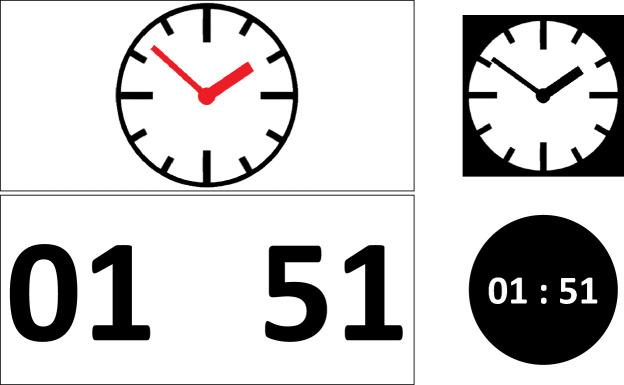
The figure shows the symbolic representation of clocks in the top row and the numerical representation in the bottom row. The size ratio between the visualizations in the HoloLens (left column, black and white inverted) and on the wristwatch (right column) equals the perceived size ratio in the real world when holding the watch at a distance of approximately 10 cm. This illustrates that some of the advantage found in the HoloLens conditions is likely to result from the possibility of displaying the information subtending a larger visual angle within the field of view.

In the clock study, we presented the time either directly in the HoloLens (without augmenting a physical clock or triggering gesture) or on a wristwatch, either using a symbolic (analogue clock face) or numerical (digital time) representation (see Fig. 3), resulting in four conditions each consisting of four training and six test trials. Here, the wristwatch conditions serve as baselines.

We presented the stimuli in the HoloLens on the physical accommodation plane of the device (2 m distance) and a height of 50 cm resulting in 14.2$^\circ$ of the visual field and locked to the center of the screen (therefore no calibration of the HoloLens was required). The wristwatch Huawei Watch 2 has a round display with a screen size of 3.05 cm, resulting in 17.3$^\circ$ of the visual field at 10 cm distance or 5.8$^\circ$ at 30 cm distance. Each stimulus was displayed until the participant gave an answer regarding the perceived time or emotion.

**Procedure.** We offered to meet with participants at the nearest station of public transport. When arriving in our lab they sat down on a chair and were introduced to the HoloLens. After filling out a consent form, we reminded the participants that they were allowed to abort the study at any point or take breaks in case they felt uncomfortable.

*Clock.* We first confirmed that the participants could perceive the clock. Participants were allowed to freely chose the distance between their eyes and the wristwatch. Afterwards, we started the study by showing the first training trial. The participant was asked to read the time aloud as soon as they were able to recognize it. They were allowed to read the time in any format they wish (e.g., “half past 2”, “two-thirty”, etc.). We recorded the accuracy of participants’ answers and their response time.

*Emotional Expressions.* First, we asked each participant to choose a mapping between the three emotions and three of the seven colors. Afterwards, we displayed all visualizations and confirmed that our assumed mapping for Emoticons and Abstract aligned with the expectations of the participant. Next, we displayed the stimuli analogue to the clock study and the participant had to name the perceived emotion. A button press displayed the next stimulus and response times and accuracy were recorded.

## Results and Discussion

We first analyse the quantitative measures of response time in seconds (RT) and the error (E). Afterwards, we discuss subjective feedback given by the participants.

*Emotional Expressions.* None of the participants made an error when naming the displayed emotions. As all participants reported not being able to recognize facial expressions in real-life settings (P1: “When really close and with good illumination, I can sometimes recognize the teeth, when somebody laughs”), all four types of representations yield an advantage in conversation and provide a benefit to users.

**Fig. 4.**
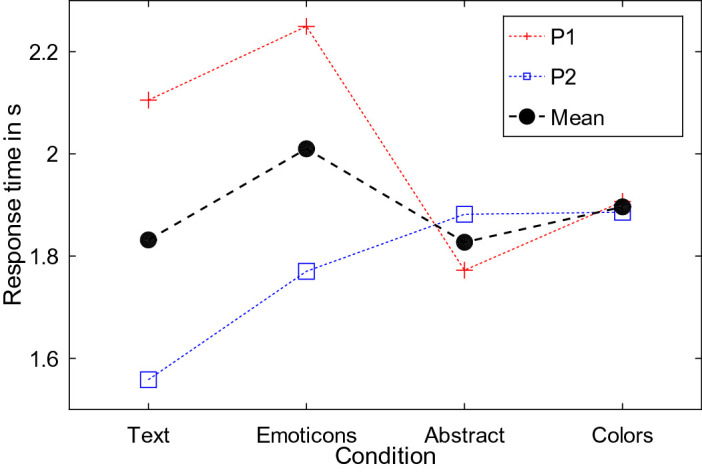
The figure shows the response times for the four visual representations of emotion. While P1 (red cross) and P2 (blue square) achieved similar results for Abstract and Colors, P1 took longer for Text and Emoticons and P2 was faster in these conditions. The mean across participants (black circle) is slightly higher for Emoticons compared to the other conditions; however, as the results of the individual participants differ substantially from each other, it is unclear whether this observation is due to the small sample size. (Color figure online)

Mean RT was highest for Emoticons (2.01 s), although both participants reported to regularly receive emoticons in instant messages. The remaining conditions had comparable RTs (Text 1.83 s; Abstract 1.83 s; Colors 1.89 s, see Fig. 4), albeit with differences in the standard deviation. Text (0.39 s) and Emoticons (0.34 s) have higher standard deviations than Abstract (0.08 s) and Colors (0.02 s). The subjective feedback provides an explanation for the larger spread. P1 stated, that Text is hard to read and he just focused on the shape of the word after reading it once during training and Emoticons are only distinguishable by the large white mouth or the red color. P2, with a higher visual acuity, reported Text to be easy to read and could recognize small details, e.g., the thin mouth, for Emoticons. We conclude that alphanumeric and complex symbolic representations are more strongly affected by the visual acuity of the participant, while simple symbolic representations are more robust.

**Fig. 5.**
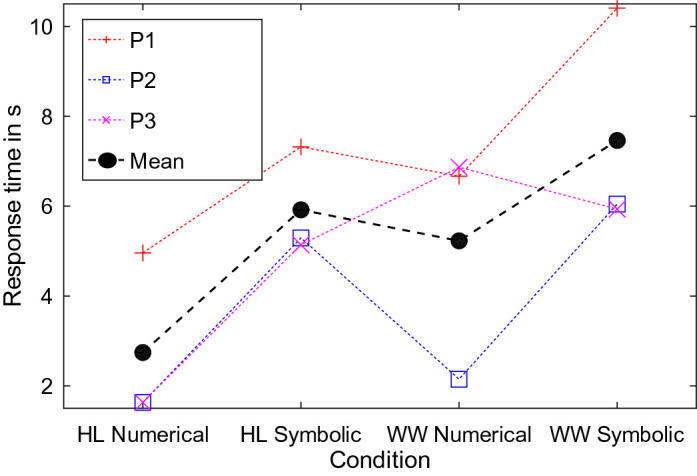
The figure shows the response times for reading the time of day for displaying the information in the HoloLens (HL) or on the wristwatch (WW). The average over all participants (black circle) shows that participants were faster using the HoloLens compared to the wristwatch and numerical compared to symbolic representation.

**Fig. 6.**
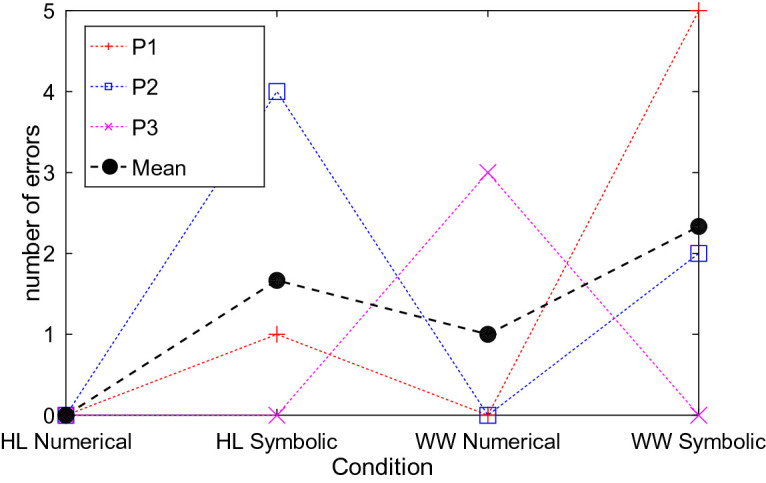
The figure shows the number of errors for reading the clock. In the condition with numerical representation, results were less error prone than in the condition with symbolic representations. Additionally, participants achieved better results with the HoloLens (HL) compared to the wristwatch (WW).

*Clock.* All participants were able to read the time both in the HoloLens and on the wristwatch. Using the HoloLens resulted in both faster and more accurate responses when determining the current time compared to the respective condition on the wristwatch (see Figs. 5 and 6). Further, responses to numerical representations were faster (HoloLens: 2.74 s vs. 5.92 s; Watch: 5.23 s vs. 7.46 s) and more accurate (HoloLens: 0.00 vs. 1.67; Watch: 1.00 vs. 2.33) than to symbolic representations. Here, the errors in the conditions with symbolic representation resulted from a misreading of the hour hand, e.g., 8:55 read as 9:55, or a mixup of the hands, e.g., 10:45 read as 8:55. Other types of inaccuracies (8:35 read as 8:30) were only observed once. All errors for numerical representations originate from visually similar digits, e.g., 8 instead of 3, or 6 instead of 5.

The participants reported preferring the AR-presentation in the HoloLens to the wristwatch. They also compared it to their usual means of obtaining the time and further confirmed some of the above findings. For instance, the advantage of the augmentation is likely attributable to the possibility of magnification and accompanying decrease in visual clutter. P1 said “The HoloLens requires less effort” and “The space between the clock hands is easier to perceive on the HoloLens”. P2 was amazed by the numerical representation in the HoloLens, “The numbers are huge, even larger than on my mobile phone, this is great”. P3 complained about the size of the numerical representation on the wristwatch “This is really small, and the numbers are very close together. This is hard for me”.

## Conclusion

Our results demonstrate that the HoloLens enables users to recognize facial expressions and further provides a benefit over a wristwatch when reading the time. Alphanumerical representations are easier to read, as long as the visual acuity of the user is sufficient to resolve the displayed text. However, if the visual acuity is too low to read even large text in the HoloLens, a symbolic representation can provide a good or even better alternative. In general, simple symbolic visualizations are more robust against differences in acuity. In conclusion, an AR system, especially if customized to the visual acuity of the user, can assist in everyday situations to individuals with low vision, either by providing access to previously non-accessible information or by helping to process available information faster.
